# Identification of subtypes and biomarkers associated with disulfidptosis-related ferroptosis in ulcerative colitis

**DOI:** 10.1186/s41065-025-00390-y

**Published:** 2025-02-22

**Authors:** Yinghao Jiang, Hongyan Meng, Xin Zhang, Jinguang Yang, Chengxin Sun, Xiaoyan Wang

**Affiliations:** 1 The First Clinical Medical College, Shandong University of Traditional Chinese Medicine, Jinan, Shandong China; 2 Shandong Provincial Hospital Affiliated to Shandong First Medical University, Jinan, Shandong China; 3 Department of Gastroenterology, Affiliated Hospital of Shandong University of Traditional Chinese Medicine, Jinan, Shandong China; 4 Staff Hospital of JIER MACHINE-TOOL GROUP CO.,LTD, Jinan, China; 5 College of Traditional Chinese Medicine, Shandong University of Traditional Chinese Medicine, Jinan, Shandong China

**Keywords:** Ulcerative colitis, Disulfidptosis, Ferroptosis, Bioinformatics

## Abstract

**Background:**

Disulfidptosis and ferroptosis are different programmed cell death modes, which are closely related to the development of a variety of diseases, but the relationship between them and ulcerative colitis (UC) is still unclear. Therefore, our study aimed to explore the molecular subtypes and biomarkers associated with disulfidptosis-related ferroptosis (DRF) in UC.

**Methods:**

We used Pearson analysis to identify DRF genes. Then, we classified 140 UC samples into different subtypes based on the DRF genes and explored the biological and clinical characteristics between them. Next, the hub genes were identified by differential analysis and WGCNA algorithms, and three machine learning algorithms were used to screen biomarkers for UC from hub genes. In addition, we analyzed the relationship between biomarkers of immune cells and transcription factors and predicted natural compounds that might be used to treat UC. Finally, we further verified the reliability of the markers by RT-qPCR experiments.

**Results:**

118 DRF genes were identified using Pearson analysis. Based on the expression level of the DRF genes, we classified UC patients into C1 and C2 subtypes, with significant differences in the abundance of immune infiltration and disease activity between the two subtypes. The machine learning algorithms identified three biomarkers, including XBP1, FH, and MAP3K5. Further analyses revealed that the three biomarkers were closely associated with a variety of immune cells and transcription factors. In addition, six natural compounds corresponding to the biomarkers were predicted, which may contribute to the effective treatment of UC. Finally, the expression trends of XBP1, FH, and MAP3K5 in animal experiments were consistent with the results of bioinformatics analysis.

**Conclusion:**

In this study, we systematically elucidated the role of DRF genes in the development of UC, and identified three potential biomarkers, providing a new idea for the diagnosis and treatment of UC.

**Supplementary Information:**

The online version contains supplementary material available at 10.1186/s41065-025-00390-y.

## Introduction

Ulcerative colitis (UC) is a chronic inflammatory disease of the colon that affects the rectum and colon to varying degrees. Its clinical manifestations often include abdominal colic during defecation, bloody diarrhea, and the discharge of pus and mucus [1]. In recent years, the incidence of UC has continued to rise in several newly industrialized countries, and UC has become a global disease with a huge economic burden [2]. The pathogenesis of UC is still unclear, and intestinal epithelial barrier defects, intestinal microecological changes, and immune dysregulation are closely related to the development of UC [3]. Currently, aminosalicylic acid, thiopurine, and biological agents are commonly used to treat UC in clinics. However, UC is prone to recurrence, and long-term treatment may lead to serious adverse effects [4]. Therefore, it is necessary to explore the molecular subtypes and new biomarkers of UC and combine precision therapy with personalized drugs to improve clinical efficacy.

Ferroptosis is a form of programmed cell death dependent on iron ions, which is caused by excessive accumulation of lipid peroxides and reactive oxygen species, leading to cell membrane damage [5]. Ferroptosis can occur through the extrinsic transporter-dependent pathway and intrinsic enzyme-regulated pathway, and it is closely associated with the pathophysiological processes in a wide range of diseases, including tumours, nervous system diseases, ischemia-reperfusion injury, kidney injury, and blood diseases [6, 7]. It has been found that ferroptosis is involved in the pathological process of inflammatory bowel disease in mice, and intestinal epithelial cell death in UC is also associated with ferroptosis [8, 9]. This suggests that ferroptosis is closely related to the development of UC.

Disulfidptosis is a novel mode of cell death triggered by disulfide stress that promotes disulfide binding in certain redox-sensitive proteins, resulting in altered protein function [10]. The researchers found that under glucose-deprived conditions, the excessive accumulation of intracellular cystine promoted the abnormal accumulation of disulfide in the cell, which in turn triggered disulfide stress, leading to abnormal disulfide bonding between actin cytoskeletal proteins, and ultimately triggered a rapid cell death, known as disulfidptosis [11]. Previous bioinformatics analysis revealed an association between disulfidptosis, ferroptosis and hepatocellular carcinoma [12, 13]. However, no study has systematically explored the relationship between disulfidptosis, ferroptosis and UC.

Therefore, in the present study, we combined disulfidptosis with ferroptosis to better elucidate the potential relationship between these two forms of cell death and UC. First, we used Pearson analysis to identify disulfidptosis-related ferroptosis (DRF) genes. Next, unsupervised cluster analysis was used to classify UC into two subtypes based on the expression of DRF genes. Then, the immune cell infiltration, inflammatory factor expression levels, and clinical features of the two subtypes were compared. Then, the hub genes were screened using differential analysis and WGCNA. In addition, we further screened the hub genes by machine learning to obtain new biomarkers and constructed a diagnostic model. Finally, the relationship between biomarkers and UC was validated through animal experiments. Our findings provide new insights for understanding the heterogeneity of UC and exploring potential drug therapy targets.

## Materials and methods

### Data sources and processing

UC gene expression profiling data were downloaded from the Gene Expression Omnibus (GEO) database (https://www.ncbi.nlm.nih.gov/geo/). The screening criteria were as follows: (1) Homo sapiens array expression profiles; (2) Intestinal mucosal tissue biopsies from healthy individuals and patients with UC; (3) Intestinal mucosal biopsy site was the colon; (4) The dataset contains more than 20 samples; (5) The dataset contains complete information about the samples. We selected the GSE38713, GSE48958, and GSE59071 datasets for the study, containing 32 normal and 140 UC samples, as shown in Table 1. The “sva” R package removed the batch effects between different datasets [14]. Principal component analysis (PCA) was used to assess the effect of batch effect removal. The GSE87466 dataset was used as the later validation dataset with 21 normal and 87 UC samples. In addition, we found 23 disulfidptosis-related genes based on the literature and then obtained a total of 259 ferroptosis-related genes from FerrDb (http://www.zhounan.org/ferrdb/legacy/operations/download.html) [11].

**Table 1:** Details for selected microarray datasets

Datasets	Platforms	Organism	Normal	UC	Country	Source tissue	Attribute
GSE38713	GPL570	Homo sapiens	13	30	Spain	colon	Test set
GSE48958	GPL6244	Homo sapiens	8	13	Belgium	colon	Test set
GSE59071	GPL6244	Homo sapiens	11	97	Belgium	colon	Test set
GSE87466	GPL13158	Homo sapiens	21	87	USA	colon	Validation set

### Identification of DRF genes

To obtain DRF genes, we matched gene expression profiling data with disulfidptosis-related genes and ferroptosis-related genes and used Pearson correlation analysis to detect the correlation between them (|cor| > 0.5, *p* < 0.05) [13]. The Pearson correlation coefficient has been widely used to analyze the correlation between genes [12, 15].

### Unsupervised clustering of UC patients

Nonnegative matrix factorization (NMF) is an efficient method for identifying different molecular patterns and is applicable to any random clustering algorithm [16]. Cluster analysis was performed using the R package “NMF” to classify UC patients into subtypes according to the expression levels of DRF genes. Subsequently, ssGSEA analysis was used to evaluate the DRFScores between different subtypes of UC [17]. Next, we used the MCPcounter algorithm from the R package “IOBR” to predict the population abundance of 10 cell types from the transcriptomic data, thereby obtaining the immune and stromal cell infiltration data for the relevant subtypes of DRF genes, and plotted box plots to show the differences in immune and stromal cell abundance between the different subtypes [18]. Then, we also analyzed inflammatory factors among different subtypes and visualized them by plotting box plots. In addition, we obtained the “c2.cp.kegg.symbols” file from the MSigDB web database and then performed GSVA enrichment analysis using the “GSVA” R package to evaluate the differences in the enriched gene sets among different subtypes [19]. Finally, we also analyzed the clinical features between different subtypes.

### Identification of differentially expressed genes (DEGs)

The genes expression levels between normal tissue and ulcerative colitis were analysed using the “limma” R package to find DEGs that met the screening criteria (|logFC| > 0.5, padj < 0.05) [20]. Then, the volcano plot of DEGs was drawn using the “ggplot2” R packages to show the distribution of DEGs.

### Weighted gene co-expression network analysis (WGCNA) and functional enrichment analysis

WGCNA enables association analysis of traits and genes to identify potential biomarkers or therapeutic targets [21]. We used WGCNA to identify significant modular genes highly associated with UC. First of all, the median absolute deviation (MAD) of each gene was calculated, and the top 5000 genes in MAD were selected for WGCNA analysis. Subsequently, the expression matrix was filtered using the goodSamplesGenes function in WGCNA, and then the scale-free co-expression network was constructed sequentially by choosing a soft threshold (power = 16, R^2^ = 0.85) according to the principle of scale-free networks and converting the adjacency matrix into a topological overlap matrix. Then, Cluster analysis was performed to identify gene modules with a minimum of 50 genes per module. A hierarchical clustering method was used to construct a dendrogram to calculate the correlation between the module feature genes and the disease phenotype, and the module with the highest correlation coefficient and the smallest P-value was defined as the disease feature module. The genes in the disease feature module were selected for subsequent analyses. The common genes of DRF genes, the disease feature module genes and DEGs were selected as hub genes for subsequent analysis, and then correlations between them were explored. In order to further confirm the potential functions of hub genes, we conducted various enrichment analyses using the “clusterProfiler” package [22]. Gene ontology (GO) analysis can explore the biological functions of hub genes.

### Screening and validation of biomarkers for UC

The obtained hub genes were further screened using machine learning algorithms to find biomarkers associated with UC. Least Absolute Shrinkage and Selection Operator (LASSO), a regression analysis algorithm that uses the R package “glmnet” to regularise the selection of variables, can identify genes that are significantly associated with different samples [23]. Support Vector Machine Recursive Feature Elimination (SVM-RFE) is a supervised machine learning technique utilizing the R package “e1071” and is widely used in classification and regression analyses [24]. It can avoid overfitting and thus identify biomarkers with high discriminatory power. Random Forest (RF), analyzed by the “randomForest” R package, can predict continuous variables and provides predictions with almost no obvious fluctuations, which is not constrained by the conditions of the variables and has higher accuracy, sensitivity, and specificity [25]. The overlapping genes of the above three algorithms were used as biomarkers. The expression levels of the final overlapping genes in the UC group and the Normal group were further analyzed and compared using the “limma” R package and visualized by drawing violin plots. Finally, the diagnostic efficacy of the biomarkers was evaluated using the receiver operating characteristic (ROC) curve [26].

### Establishment of a nomogram scoring system and evaluation of immune cell infiltration

Firstly, a UC diagnostic model based on biomarkers was established using the “rms” R package. Using a nomogram scoring system, the relative expression level of each gene corresponds to a score, and the sum of the scores is called the total score, which can be used to predict the incidence of UC [27]. Then, the predictive power of the line graph model was evaluated using calibration curves. The clinical application value of the model was evaluated using Decision Curve Analysis (DCA) [28]. In addition, the correlation between biomarkers and immune cell infiltration was analyzed using CIBERSORT algorithm and “corrplot” package [29].

### Prediction of transcription factors (TFs) and natural compounds

The NetworkAnalyst platform (https://www.networkanalyst.ca/) can provide insight into various mechanisms and roles of organisms by analyzing gene expression data [30]. We used the NetworkAnalyst platform to predict relevant TFs for biomarkers from the JASPAR database and visualized them using Cytospace software. The Hit 2.0 database is a comprehensive search and sorting platform for herbal ingredients and target information based on literature evidence [31]. We used the Hit 2.0 database (http://www.badd-cao.net:2345/) to predict potential natural compounds related to biomarkers.

### Animal models of UC

Animal experiments were approved by the Animal Ethics and Experimentation Committee of Shandong University of Traditional Chinese Medicine and conducted in accordance with the Guidelines for the Care and Use of Laboratory Animals (approval number: SDUTCM20230404001). Twelve C57BL/6J male mice (7 weeks old, 20–22 g) were provided by Beijing Vital River Laboratory Animal Technology Co., Ltd., China (SCXK (Jing) 2021-0006), and were fed on a standard diet under the controlled environment with 22–24 °C, 50-70% relative humidity, and a 12 h light-dark cycle. After seven days of adaptive feeding, the mice were randomly divided into the Normal group and the DSS group (*n* = 6). Mice in the Normal group freely drank purified water, and mice in the DSS group were given 3% fresh DSS solution for free drinking. After the DSS-induced UC models were successfully established, the mice were anesthetized with isoflurane gas, and colon tissues were taken out for further detection.

### Histological analysis of colon

Colon tissues of mice were fixed with 4% paraformaldehyde for 48 h, dehydrated with ethanol, and then embedded in paraffin for sectioning (thickness 4 μm). After baking at 60 °C, the sections were dewaxed and washed and stained with hematoxylin and eosin. Finally, the sections were observed and photographed with a upright white light microscope (Nikon, Japan).

### RT-qPCR

Total RNA was extracted from the colon tissues using TRIzol reagent and reverse transcribed to cDNA using the Reverse Transcription Kit. The RT-qPCR method was as follows: pre-denaturation at 95 °C for 10 min, denaturation at 95 °C for 20 s, annealing at 60 °C for 30 s, elongation at 72 °C for 20 s, 40 cycles. The sequences of primers are detailed in Table 2. The data were calculated using the 2^−ΔΔCT^ method, with β-actin as the internal reference.

**Table 2:** Primer sequences

Target genes	Primer sequence (5’-3’)
XBP1	FORWARD: TGGAGCAGCAAGTGGTGGAT REVERSE: GTCCATTCCCAAGCGTGTTC
MAP3K5	FORWARD: ACACCAGCAGCAGTAGCGAGTA REVERSE: GCCCAGGAACAATGTCCGTAT
FH	FORWARD: GCCAATCCCAGTCATTCAAGC REVERSE: TCCAGTCTGCCAAACCACCA

### Statistical analysis

Experimental data were expressed as mean ± standard deviation. The independent samples t-test was used if the data conformed to a normal distribution. The non-parametric rank sum test was used if the data didn’t conform to a normal distribution. Statistical analyses and plotting were performed using R software (version 4.2.3) and SPSS software (version 25.0). P value < 0.05 was considered statistically significant.

## Results

### Data processing

**Fig. 1:**
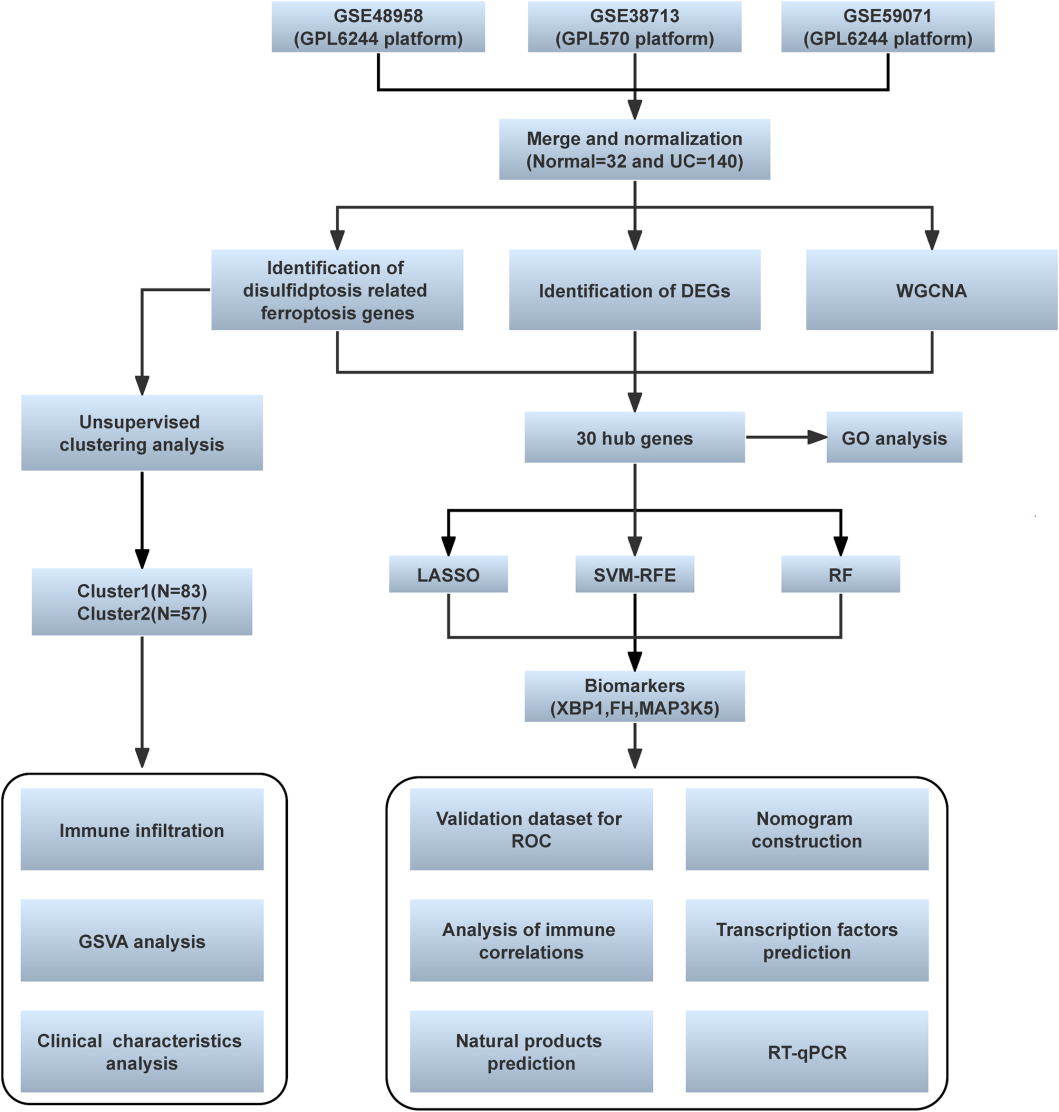
Flow chart of the study

**Fig. 2:**
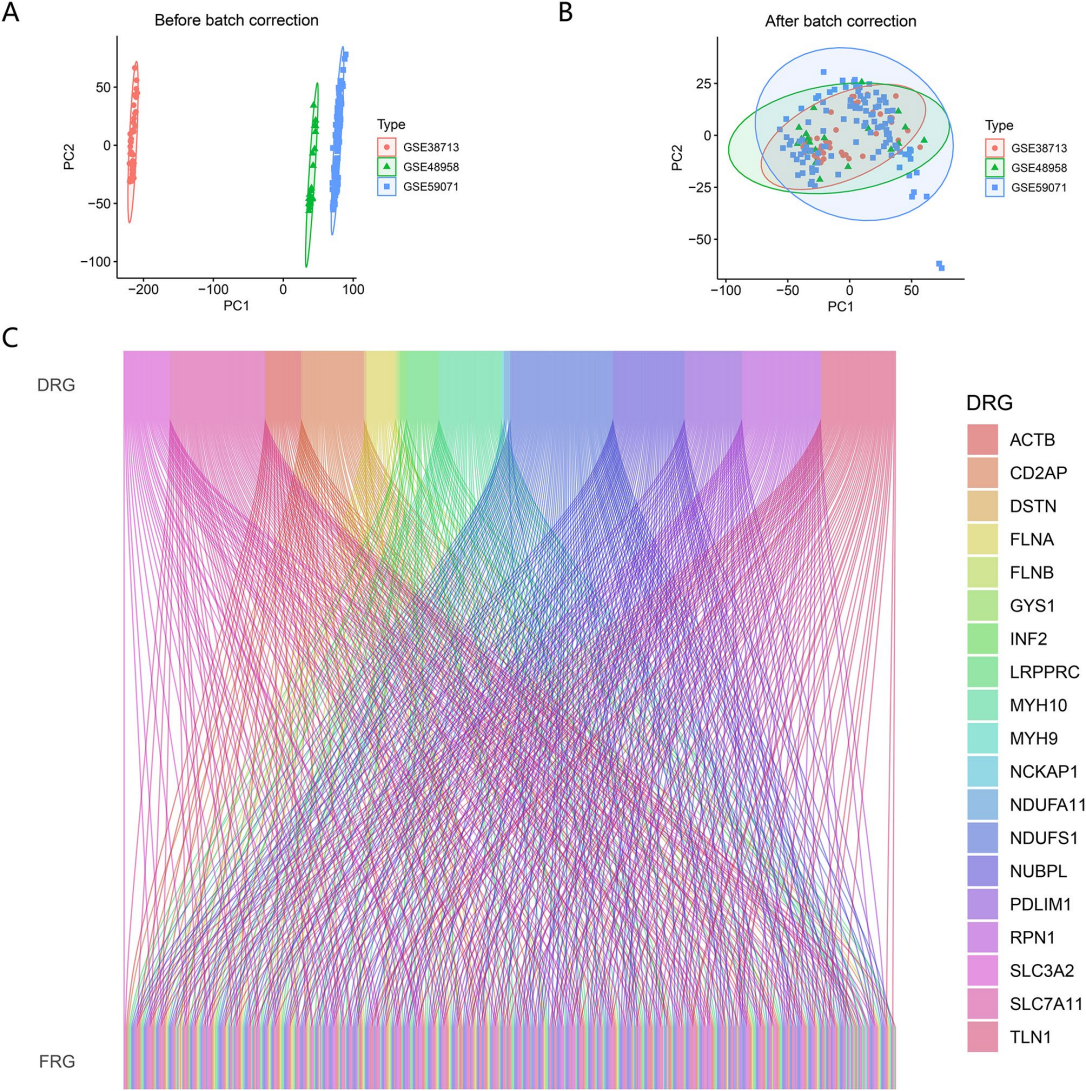
DRF genes identification. PCA showing the expression distribution of three UC datasets before (**A**) and after (**B**) batch effect removal. (**C**) The Sankey diagram of showing the correspondence between 19 DRGs and 118 FRGs. DRG: disulfidptosis-related gene; FRG: ferroptosis-related gene

The detailed study design is shown as a flow chart in Fig. 1. We downloaded four datasets from the GEO database, merged 172 samples from GSE38713, GSE48958, and GSE59071 for research, and then removed the batch effects between them using the “ComBat” function in the “sva” R package. The PCA plot shows the data distribution before and after removing the batch effect, and the results show that the batch effect is effectively removed (Fig. 2A, B). The GSE87466 dataset was used for subsequent validation. A total of 118 DRF genes were identified by correlation analysis and visualized using the Sankey diagram, which showed a close correlation between disulfidptosis genes and ferroptosis genes (Fig. 2C).

### Unsupervised clustering of UC patients

**Fig. 3:**
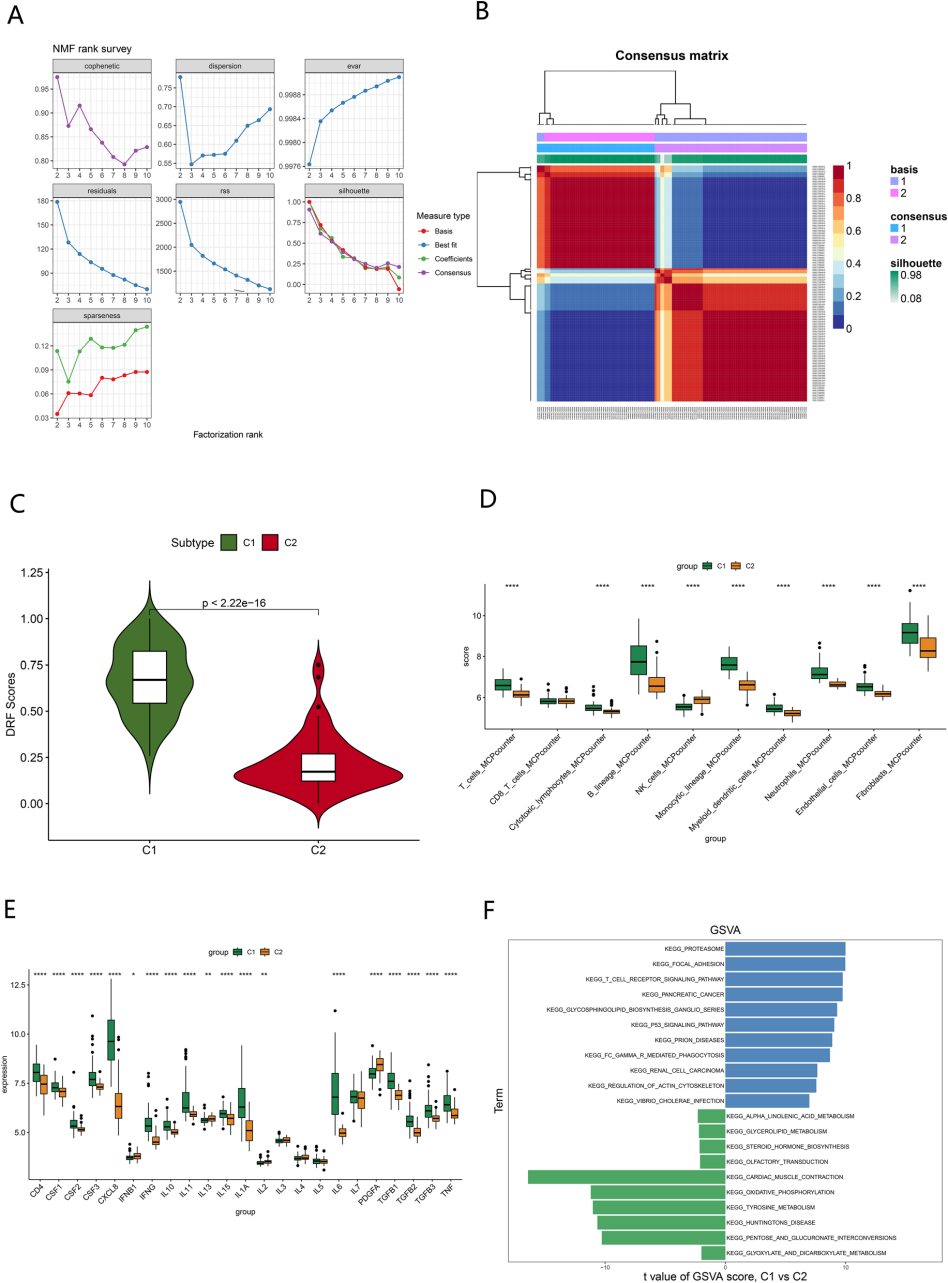
Identification of UC subtypes and exploration of biological features between the subtypes. (**A**) The factorization rank diagram of the UC subtype. (**B**) The clustering results using k = 2 is shown for UC samples. (**C**) Boxplots showed the estimated DRF score between the UC subtypes. (**D**) Boxplots showed the differences in immune infiltrating between the subtypes. (**E**) Boxplots showed the expression levels of inflammatory factors between the subtypes. (**F**) Differences in hallmark pathway activities between DRFScore_LOW and DRFScore_high samples ranked by t-value of GSVA method. *p < 0.05,**p < 0.01,****p < 0.0001

**Fig. 4:**
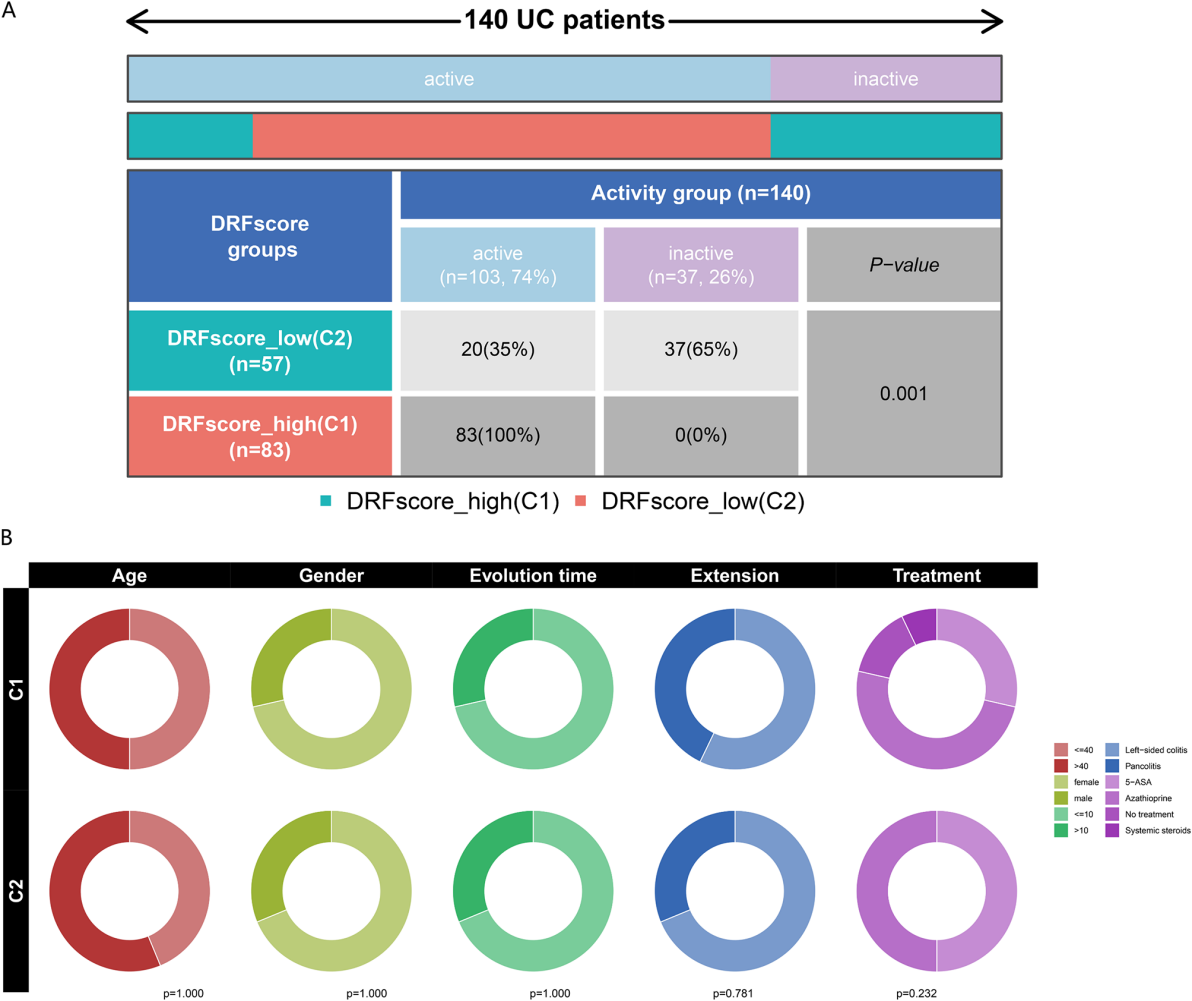
Identification of clinical features between the subtypes. (**A**) The distribution of active and inactive patients in different subtypes. (**B**) Differences in clinical features between the subtypes

Firstly, we used the R package “NMF” to classify UC patients into different subtypes based on the expression levels of 118 DRF genes. Based on the cophenetic, dispersion, and silhouette metrics, the optimal number of subtypes was chosen to be 2 (Fig. 3A). One hundred and forty UC samples were divided into two subtypes, C1 and C2 (Fig. 3B). By ssGSEA analysis, C1 was identified as the DRFScore_high (*N* = 83) and C2 as the DRFScore_low group (*N* = 57) (Fig. 3C). Then, the MCPcounter algorithm in the “IOBR” R package was used to estimate the abundance of tissue-infiltrating immune cells and stromal cell populations. The boxplot showed that the abundance of DRFScore_high was higher than that of DRFScore_low in T cells, cytotoxic lymphocytes, B lineage, monocytic lineage, myeloid dendritic cells, neutrophils, endothelial cells, and fibroblasts, while the abundance of DRFScore_high was lower than that of DRFScore_low in NK cells (Fig. 3D). Next, the statistical analysis was conducted on the expression levels of inflammatory factors between the two groups. The results showed that the expression levels of CD4, CSF1, CSF2, CSF3, CXCL8, IFNG, IL10, IL11, IL15, IL1A, IL6, TGFB1, TGFB2, TGFB3, and TNF were lower in DRFScore_low and higher in DRFScore_high. IFNB1, IL-13, and PDGFA were highly expressed in DRFScore_low but lowly expressed in DRFScore_high (Fig. 3E). The GSVA analysis revealed that the P53 signaling pathway and T cell receptor signaling pathway were upregulated in the DRFScore_high group, whereas oxidative phosphorylation and tyrosine metabolism were upregulated in the DRFScore_low group (Fig. 3F). Finally, a comparative analysis of the clinical characteristics between the different subtypes showed that the DRFScore_high group was all patients with active UC, while the DRFScore_low group was mainly patients with inactive UC (Fig. 4A). In addition, there was no significant difference in age, gender, evolution time, extension, and treatment between DRFScore_high and DRFScore_low (Fig. 4B).

### **Identification of DEGs and WGCNA analysis**

**Fig. 5:**
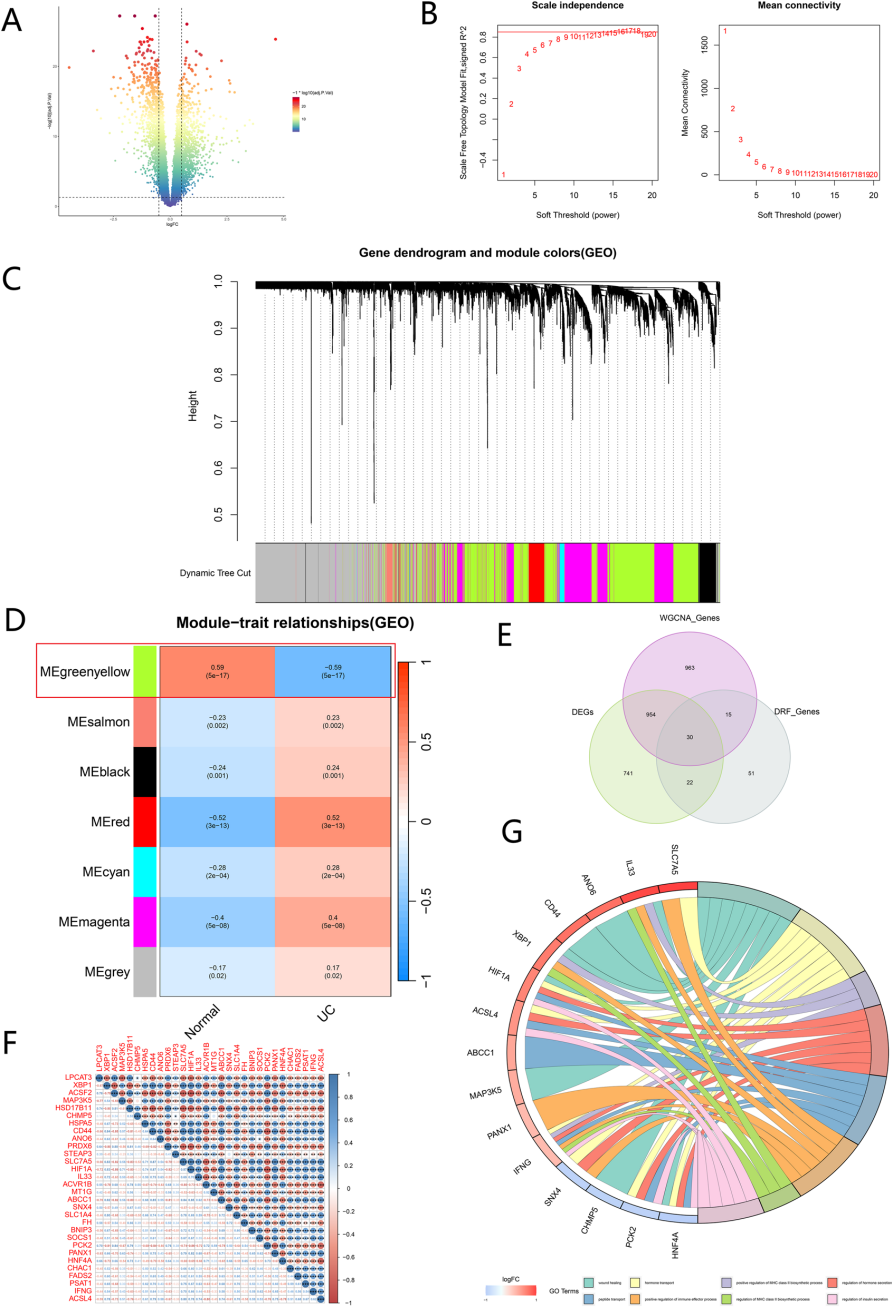
DEGs and WGCNA analysis. (**A**) Volcano plot showing DEGs between the UC groups and the Normal groups. (**B**) Scale-free fit index and network connectivity under different soft thresholds. (**C**) Gene hierarchy tree clustering diagram. (**D**) Heatmap showing the relations between the module and UC features. (**E**) Venn diagram of DRF genes, WGCNA genes, and DEGs. (**F**) Comparison of 30 hub genes between the UC group and the Normal group. Red and blue represent positive and negative correlations, respectively. The correlation coefficient is shown as the circle of the area of the circle. (**G**) The results of GO enrichment analysis

Differential analysis of gene expression profiling data between normal and UC groups was performed using the “limma” R package. As shown in the volcano diagram (Fig. 5A), we obtained 1747 DEGs.

To further explore the core genes related to the UC phenotype, we constructed a gene co-expression network using the WGCNA algorithm. Firstly, the top 5000 genes with Median Absolute Deviation were screened out for analysis. Subsequently, we evaluated the scale-free fit index and average connectivity of various soft-threshold powers based on scale-free R^2^. A soft threshold power of 16 with scale-free R^2^ of 0.85 was selected for our study to construct a standard scale-free network with a Pick Soft Threshold function (Fig. 5B). A gene hierarchical clustering tree diagram was constructed by gene association, and seven consensus co-expression modules were identified (Fig. 5C). The heatmap of the correlation between module feature values and UC phenotype showed that the genes in the greenyellow module were highly correlated with UC (*R* = 0.59, *P* = 5e-17) (Fig. 5D). Therefore, the genes in the greenyellow module were selected for further analysis. A total of 30 hub genes were obtained by intersecting DRF genes, WGCNA-screened genes, and DEGs (Fig. 5E). Correlation analyses revealed that most hub genes were closely related, except for CHMP5, MAP3K5, and MT1G (Fig. 5F).

Furthermore, we conducted GO enrichment analysis on the 30 hub genes obtained. The results of GO enrichment analysis indicated that core genes were mainly involved in two healing, hormone transport, positive regulation of MHC class II biological process, regulation of hormone secretion, peptide transport, positive regulation of immune effector process, regulation of MHC class II biological process and regulation of insulin secretion (Fig. 5G).

### Identification of biomarkers in UC

**Fig. 6:**
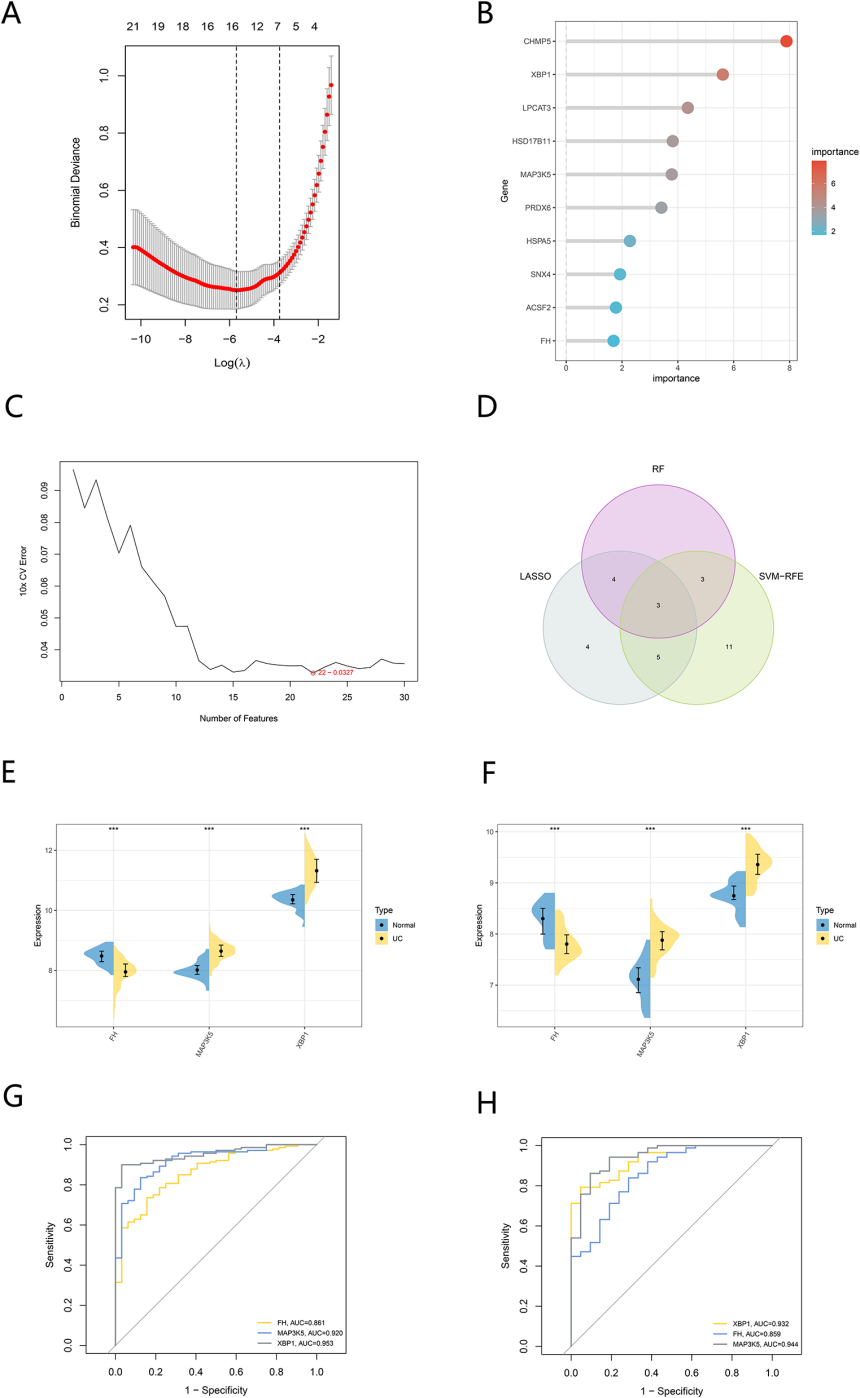
Identification of biomarkers. (**A**) LASSO logistic regression was used to screen potential biomarkers. (**B**) Identifying potential biomarkers using RF algorithm. The x-axis indicates the gene, and the y-axis represents the importance index. (**C**) Screening of potential biomarkers for diseases based on SVM-RFE algorithm. (**D**) The Venn diagram shows the overlapping genes between the above three algorithms. (**E**, **F**) The expression of the three biomarkers in test set (**E**) and validation set (**F**). (**G**, **H**) ROC curve plot of marker genes in test set (**I**) and validation set (**J**). ***p < 0.001, **p < 0.01,*p < 0.05, Normal vs. UC group

After obtaining 30 hub genes, we used three machine algorithms to screen feature genes from them as biomarkers for UC. Using the LASSO regression algorithm, 16 genes were selected as potential biomarkers (Fig. 6A). In the RF algorithm, we selected the genes with the top 10 importance values as candidate genes for further analysis (Fig. 6B). The SVM-RFE algorithm showed the highest accuracy when the number of feature genes was 22 (Fig. 6C). Ultimately, we took the intersection of the feature genes obtained by the above three methods to obtain three overlapping genes as biomarkers for UC (Fig. 6D). The violin plot showed the expression of three genes in the merged GEO dataset (Fig. 6E, F). The results showed that the expression levels of MAP3K5 and XBP1 of UC patients were higher than those of normal patients, while the expression levels of FH were lower than those of normal patients. The above results were also confirmed in the validation dataset GSE87466. This suggests that they may play a role in the occurrence and development of UC. The ROC analysis was used to further validate the diagnostic value of FH, MAP3K5, and XBP1 (Fig. 6G, H). In the merged GEO dataset, the AUC values of the ROC curves for all three genes exceeded 0.8, indicating that these genes were diagnostic of UC. In addition, the AUC values of these three overlapping genes in the independent patient cohort of the GSE87466 dataset were more than 0.8, suggesting that FH, MAP3K5, and XBP1 had a high diagnostic value.

### Nomogram construction of UC diagnosis model based on biomarkers and immune infiltration analyses

**Fig. 7:**
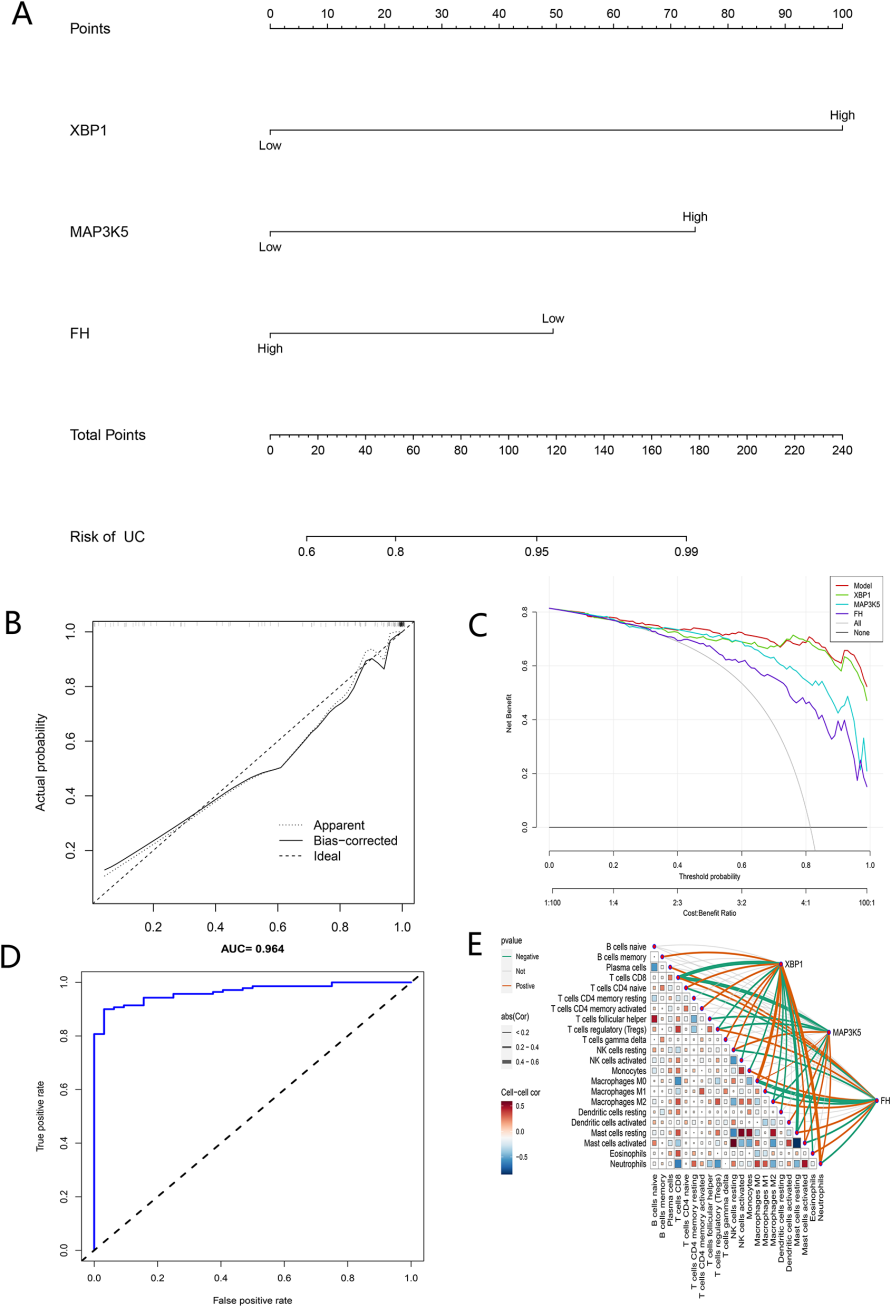
Construction of the UC diagnostic model and immune infiltration analyses. (**A**) Nomogram of the UC diagnostic model based on the three biomarkers. (**B**) Calibration curves for assessing the predictive power of the model. (**C**) DCA curves to assess the clinical value of the model. (**D**) The ROC curves were used to evaluate the diagnostic efficacy of the UC diagnostic nomogram. (**E**) The association between the three biomarkers and immune cell infiltration

Based on the expression levels of the three overlapping genes, we constructed a UC diagnostic model based on logistic regression and drew a nomogram (Fig. 7A). In the nomogram, the expression of each gene participating in the construction of the diagnostic model corresponded to a score, and their scores were added to obtain the total score, which corresponded to the different incidence rates of UC. The calibration curves showed that the difference between the true and predicted risk of UC was very small, indicating that the nomogram could diagnose UC reliably (Fig. 7B) In the DCA, the nomogram provided greater clinical benefit than the “FH, MAP3K5, and XBP1” curve (Fig. 7C). The AUC of the nomogram’s ROC curve was 0.96, which also demonstrated the reliability of the diagnostic model (Fig. 7D).

The correlation between the expression levels of three biomarkers and immune cell infiltration in UC was investigated by the CIBERSORT algorithm. The results showed that the expression of FH was positively correlated with the abundance of Macrophages M2, and negatively correlated with the abundance of Macrophages M0. The expression of XBP1 was positively correlated with the abundance of NK cells resting, and negatively correlated with the abundance of NK cells activated. The MAP3K5 expression was positively correlated with the abundance of Macrophages M0, and negatively correlated with the abundance of Macrophages M2 (Fig. 7E).

### Determination of TFs and potential natural compounds

**Fig. 8:**
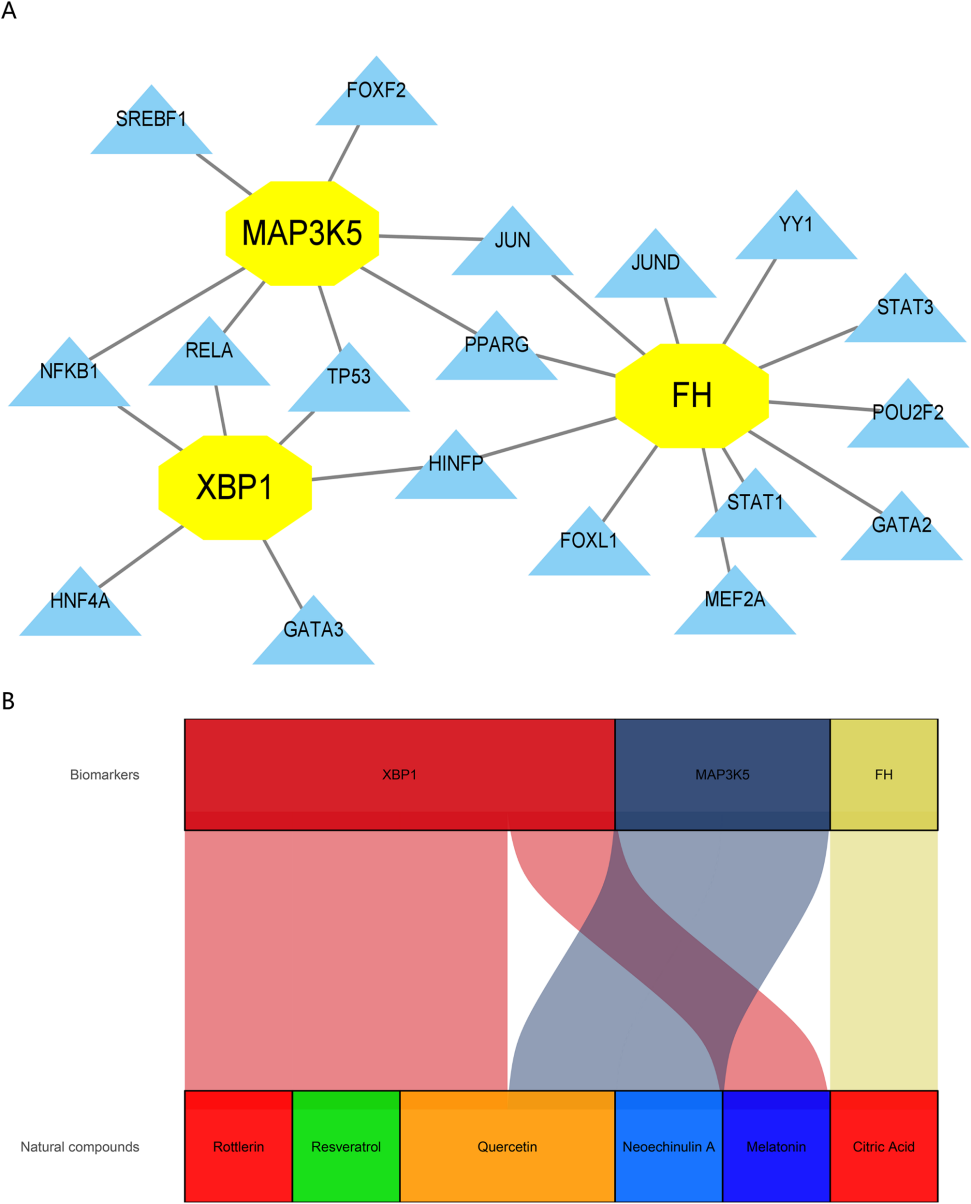
Prediction of transcription factors and natural compounds. (**A**) The TFs-genes network. (**B**) The Sankey diagram revealed the relationship between biomarkers and natural compounds

Through the NetworkAnalyst platform, we found that XBP1 is regulated by 6 TFs, MAP3K5 by 7 TFs, and FH by 11 TFs (Fig. 8A). In addition, we predicted the potential natural compounds corresponding to the three biomarkers using the HIT 2.0 database to obtain six natural compounds that might be used for the treatment of UC, and the results are shown in the Sankey diagram (Fig. 8B).

### Validation in animal models

**Fig. 9:**
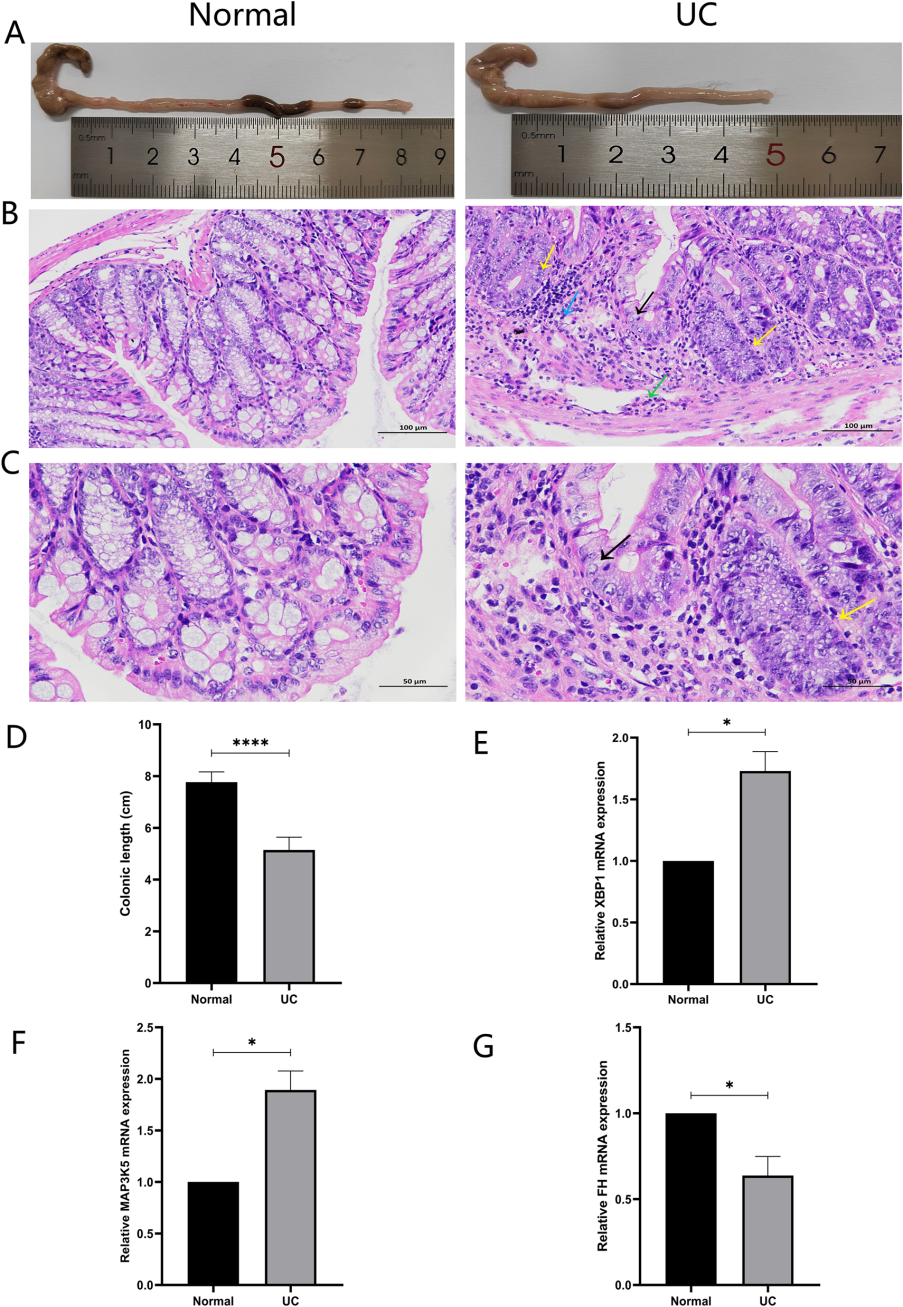
The results of animal experiments. (**A**) Representative images of colons from each group. (**B**, **C**) Representative images of H&E staining of colonic mucosa in both groups. (Blue arrow: lymphocyte infiltration; Yellow arrow: intestinal gland atypia; Green arrow: granulocytic infiltration; Black arrow: mucosal epithelial cell necrosis; *n* = 3). (**B**) magnification = 200×, scale bar 100 μm. (**C**) magnification = 400×, scale bar 50 μm. (**D**) The lengths of colons of each group of mice (*n* = 6). (**E**-**G**) The mRNA expression levels of three biomarkers in colon tissues (*n* = 3). *p < 0.05, ****p < 0.0001

To further explore the diagnostic value of these three biomarkers for UC, we validated them in the UC model of C57BL/6 mice. The research found that the colons of the DSS group were congested and swollen, with bloody stools, and the lengths of the colons were significantly shorter than those of the Normal group (Fig. 9A, D). Histological analysis showed that the colon of mice in the DSS group was extensively ulcerated, with necrosis of mucosal epithelial cells, infiltration of lymphocytes in the lamina propria, a few intestinal gland atypia, and infiltration of granulocytes in the submucosal layer. (Fig. 9B, C). All of the above suggested that the UC model of mice had been successfully established. Furthermore, we detected the mRNA expression levels of FH, MAP3K5, and XBP1 in the colons of mice. The results showed that the expression levels of MAP3K5 and XBP1 were significantly higher and the expression level of FH was significantly lower in the DSS group compared with the Normal group (Fig. 9E-G).

## Discussion

UC is a lifelong inflammatory disease that can severely affect the quality of life of patients [32]. Although the treatment methods for UC are constantly increasing, it still cannot be eradicated, and the adverse effects caused by long-term treatment cannot be ignored [33, 34]. Therefore, there is an urgent need to understand the heterogeneity of UC and explore biomarkers to provide more precise treatment for patients.

Many previous studies have found that ulcerative colitis is associated with a variety of regulatory cell deaths, such as autophagy, pyroptosis, and apoptosis [35–37]. However, the role of disulfidptosis and ferroptosis in UC remains to be elucidated. Therefore, this study combined the two for a more comprehensive analysis, aiming to explore the molecular subtypes and biomarkers of UC based on the characteristics of disulfidptosis and ferroptosis, and further clarify the relationship between these two cell death modes and the pathogenesis of UC.

In this study, we first explored the correlation between 23 disulfidptosis-related genes and 259 ferroptosis-related genes by analyzing gene expression profiling data and obtained 118 DRF genes. Then, the DRF-related UC molecular subtypes were constructed by unsupervised clustering analysis, and the UC samples were divided into two groups: the DRFScore_high group and the DRFScore_low group. The MCPcounter algorithm showed that DRFScore_high exhibited relatively high levels of immune infiltration. In order to investigate the inflammatory features between the two subtypes, we analyzed the expression levels of inflammatory factors, and the results showed that the inflammatory levels of DRFScore_high were relatively high. Clinical features analyses showed the DRFScore classification was related to disease activity and not clinical traits such as age and gender. Therefore, we conclude that high expression of DRFScore in UC indicates higher levels of immune infiltration and inflammation. Notably, DRFScore_high was predominantly enriched in the P53 signaling pathway, T cell receptor signaling pathway, both of which can promote intestinal inflammation [38, 39]. Some studies have also shown that active UC has higher levels of immune infiltration and inflammation compared to inactive UC [40, 41]. The above results indicate that disulfidptosis and ferroptosis can affect the immune microenvironment of UC and promote the inflammatory response, thus aggravating the existing condition.

Subsequently, 30 hub genes were screened by differential analysis and WGCNA analysis, and correlation analysis showed that the roles of these hub genes in UC were significantly consistent. Go analysis showed that the 30 core genes were mainly enriched in the regulation of MHC class II biological process, regulation of hormone secretion, peptide transport and positive regulation of immune effector process. The above biological processes are closely related to the occurrence and development of UC [42–45].

Due to the high precision and low error rate of machine learning in screening key genes, it is increasingly used to find biomarkers to explore new therapeutic targets [46, 47]. Therefore, we screened out three potential biomarkers for UC, XBP1, MAP3K5, and FH, using LASSO, SVM-RFE, and RF machine learning algorithms. XBP1 is a protein-coding gene that plays a transcription factor role by regulating the unfolded protein response during endoplasmic reticulum stress [48]. Arthur and colleagues found that aberrant expression of XBP1 in intestinal epithelial cells induces endoplasmic reticulum stress, which leads to intestinal inflammation, suggesting that XBP1 expression is strongly associated with developing inflammatory bowel disease [49]. It has also been found that XBP1 is also associated with the immunosuppressive function of M2 cells in UC. It impairs immunosuppressive function by promoting the expression of ring-finger protein 20 and decreasing the abundance of PD-L1 in M2 cells [50]. FH is an enzyme in the tricarboxylic acid cycle that catalyzes the hydration of fumaric acid to malic acid in both mitochondria and cytoplasm [51]. Recently, it has been found that decreased FH expression inhibits interleukin-10 expression, leading to increased expression of TNF and a strong inflammatory response [52]. TNF-α is highly expressed in the intestinal tissues of UC patients, suggesting that FH may also have potential pathogenic effects in UC [53]. MAP3K5, also known as ASK1, is an important component of the MAP kinase signaling pathway, which can regulate various cellular responses such as apoptosis, differentiation, and so on [54, 55]. The activity of MAP3K5 can be triggered by various stressors such as endoplasmic reticulum stress, calcium influx, and reactive oxygen species stress, and its dysregulation is related to the development of various diseases [56]. In addition, the Calcium-ASK1 signaling cascade has been reported to be associated with DSS-induced disruption of intestinal epithelial tight junctions, which suggests that MAP3K5 may be involved in the pathogenesis of UC [57]. We found that the AUC values of the above three genes were greater than 0.8 in both the combined dataset and the validation dataset, indicating that they have good diagnostic value for UC and may be potential targets for treating UC. Subsequently, we constructed a nomogram model for diagnosing UC based on XBP1, MAP3K5, and FH, which can calculate the risk of UC according to the expression levels of these three biomarkers in patients, which is of great significance for the early diagnosis of UC. By using the nomogram, physicians can calculate the disease risk more conveniently and directly, and then make clinical decisions. Although the calibration and ROC curves also indicate that the model has high accuracy and reliability in diagnosing UC, more clinical samples need to be collected to validate its clinical utility and improve the stability and clinical applicability. To further explore the roles of these three biomarkers in immune regulation, we analyzed them using the CIBERSORT algorithm and found that XBP1 was associated with NK cells, MAP3K5 and FH were associated with macrophages. It has been found that XBP1 plays an important role in controlling NK cell immunity, which can promote the production of IFN-γ by interacting with T-bet, thereby enhancing the effector function of NK cells [58]. Down-regulation of FH leads to accumulation of fumaric acid in the cytoplasm, which regulates the production of cytokine, such as IL-10 and IFNs, leading to macrophage activation [59]. MAP3K5 is required for the innate immune response, and it has been found that ASK1-K716 can modulate the polarization of macrophages and downregulate the expression of pro-inflammatory factors, thereby alleviating early neuroinflammation [60].

TFs, which bind DNA in a sequence-specific manner and regulate transcription, underlie many aspects of human physiology, disease, and variation [61]. Our predicted TFs, such as NFKB1, PPARG, and RELA, are closely related to the development of UC [62, 63]. In addition, six natural compounds that may be used in the treatment of UC were identified in the HIT 2.0 database. Quercetin, melatonin, and resveratrol have been shown to alleviate UC somewhat. Quercetin is a flavonoid compound with pharmacological activities such as anti-inflammatory and antioxidant, and it has been found that quercetin can alleviate UC by repairing intestinal barrier dysfunction and regulating the selective activation of macrophages [64, 65]. Melatonin is an amine hormone with immune and metabolic regulatory functions, which may improve UC by reducing inflammatory damage and restoring colonic damage [66, 67]. Resveratrol is a non-flavonoid polyphenol organic compound with antioxidant and anti-inflammatory effects. Studies have found that it can affect the intestinal microbiota, inhibiting autophagy, and reduce the expression of inflammatory factors, thereby ameliorating DSS-induced UC [68, 69]. Previous clinical studies have also demonstrated that quercetin, melatonin, and resveratrol can improve the quality of life of patients with UC and reduce the inflammatory response in patients with UC [70–72]. However rottlerin, citric acid, and neoechinulin A have no evidence to treat UC at this time.

In animal experiments, compared with the normal mice, the mRNA levels of XBP1 and MAP3K5 in the intestinal tissues of UC mice were significantly increased, while the mRNA levels of FH were decreased considerably. These findings suggest that changes in the mRNA levels of these three genes may be related to the development of UC, providing a basis for exploring new diagnostic and therapeutic methods for UC. However, this study also has certain limitations. Firstly, because our data come from the public database, we still need to collect more clinical data and use methods such as deep learning for more in-depth clinical validation. Secondly, although we have verified gene expression in mice, it is still necessary to explore deeper mechanisms through in vitro or in vivo experiments with human samples.

## Conclusion

Through comprehensive and systematic bioinformatics analyses, we revealed the potential roles of disulfidptosis and ferroptosis in UC and further elucidated the clinical and immunological heterogeneity among different DRF subtypes in UC patients. In addition, combining multiple machine learning algorithms, we screened the biomarkers XBP1, MAP3K5, and FH, established a diagnostic model containing these three biomarkers, explored their relationship with immune cells, and predicted several natural compounds with the potential to treat UC. This study lays the foundation for further research on the pathogenic mechanism of UC and provides new ideas for early diagnosis and precise treatment of UC.

## Electronic supplementary material

Below is the link to the electronic supplementary material.

Supplementary Material 1

Supplementary Material 2

Supplementary Material 3

Supplementary Material 4

Supplementary Material 5

Supplementary Material 6

Supplementary Material 7

## Data Availability

Data pertinent to this study are available upon request via email to the corresponding author.
